# Bioresorbable Polymeric Scaffold in Cardiovascular Applications

**DOI:** 10.3390/ijms21103444

**Published:** 2020-05-13

**Authors:** Daniel Wee Yee Toong, Han Wei Toh, Jaryl Chen Koon Ng, Philip En Hou Wong, Hwa Liang Leo, Subramanian Venkatraman, Lay Poh Tan, Hui Ying Ang, Yingying Huang

**Affiliations:** 1 School of Materials Science and Engineering, Nanyang Technological University, Nanyang Avenue, Singapore 639798, Singapore; DTOONG001@e.ntu.edu.sg (D.W.Y.T.); lptan@ntu.edu.sg (L.P.T.); 2 National Heart Centre Singapore, 5 Hospital Drive, Singapore 169609, Singapore; hanweitohh@gmail.com (H.W.T.); jaryl.ng.c.k@nhcs.com.sg (J.C.K.N.); philip.wong.e.h@singhealth.com.sg (P.E.H.W.); 3 Department of Biomedical Engineering, National University of Singapore, 4 Engineering Drive 3, Singapore 117583, Singapore; bielhl@nus.edu.sg; 4 Duke-NUS Medical School, National University of Singapore, 8 College Road, Singapore 169857, Singapore; 5 Materials Science and Engineering, National University of Singapore, 9 Engineering Drive 1, Singapore 117575, Singapore; subbu@nus.edu.sg

**Keywords:** bioresorbable scaffolds, biomaterials, polymeric scaffolds, cardiovascular tissue engineering, vascular grafts, cardiac patches

## Abstract

Advances in material science and innovative medical technologies have allowed the development of less invasive interventional procedures for deploying implant devices, including scaffolds for cardiac tissue engineering. Biodegradable materials (e.g., resorbable polymers) are employed in devices that are only needed for a transient period. In the case of coronary stents, the device is only required for 6–8 months before positive remodelling takes place. Hence, biodegradable polymeric stents have been considered to promote this positive remodelling and eliminate the issue of permanent caging of the vessel. In tissue engineering, the role of the scaffold is to support favourable cell-scaffold interaction to stimulate formation of functional tissue. The ideal outcome is for the cells to produce their own extracellular matrix over time and eventually replace the implanted scaffold or tissue engineered construct. Synthetic biodegradable polymers are the favoured candidates as scaffolds, because their degradation rates can be manipulated over a broad time scale, and they may be functionalised easily. This review presents an overview of coronary heart disease, the limitations of current interventions and how biomaterials can be used to potentially circumvent these shortcomings in bioresorbable stents, vascular grafts and cardiac patches. The material specifications, type of polymers used, current progress and future challenges for each application will be discussed in this manuscript.

## 1. Introduction

The term cardiovascular disease (CVD) is used to describe a group of diseases that afflicts the structure and function of the heart or blood vessels [1], with the coronary heart disease (CHD) subset being responsible for the greatest cause of cardiovascular deaths. While considerable progress in the treatment and management of CVD has resulted in the 50% reduction in CVD related mortality in the 1970s [1,2], CVD still remains as a major contributor of death and disability globally [2]. According to the World Health Organisation, an estimated 17.9 million people have died from CVD, which represented 31% of deaths globally in 2016 [3]. As CHD occurs through a deposition of atherosclerotic plaque on the arterial wall, it limits the blood flow to myocardium that can leading to cardiac ischemia. As a preventive measure for patients with 50% to 70% stenosis, the usage of stents can be deployed to the site of lesion to ‘open up’ the stricture to restore blood flow [4]. However, a permanent stent in the long run can eventually result in a late catch-up phenomenon, while the previously FDA-approved bioresorbable stent faced issues with its high strut thickness that translates to further complications. In situations where stents cannot be deployed in arteries due to high SYNTAX score levels (≥34), coronary artery bypass graft (CABG) will be performed with a saphenous vein graft to reroute the flow of blood to the heart [5]. However, the suitability of the vein graft and other artificial conduits may not be ideal due to incompatible size and properties mismatch (such as compliance, stiffness) which can lead to thrombosis [6]. In cases where patients have experienced prior myocardial infarction (MI), the heart muscles are damaged and thinned [7]. This will in turn result in inefficient pumping of the heart and can lead to complications such as cardiac arrythmias.

The advancement in material science and innovative medical technologies has allowed the application of less invasive percutaneous treatment of CHD in order to address the shortcomings of current options. Furthermore, the advent of tissue engineering and regenerative medicine spurred R&D efforts in producing treatment options such as bioresorbable stents, vascular grafts and cardiac patches. This review paper will focus on the progress and current development of bioresorbable polymers in cardiovascular applications, either for use in interventional therapy or as a platform for regenerative medicine.

### Use of Biomaterials in the Treatment of CHD

The definition of biomaterial was first defined in 1987 as ‘a non-viable material used in a medical device, intended to interact with biological systems’, the definition subsequently evolved to ‘a material intended to interface with biological systems to evaluate, treat, augment or replace any tissue, organ or function of the body’ [8]. The evolution in definition reflected the developmental progress of biomaterials; from being biologically inert to being bioactive and biocompatible. Traditionally, materials can be characterised into three primary types: metallic, ceramic and polymeric [9]. These three primary types were differentiated by the bonds that are present within the substance, with metals possessing metallic bonds, ceramics bonded by ionic interactions and polymers having covalent bonds [9]. Biomaterials used in biomedical applications will usually involve these three primary types with polymers and metals used commonly in cardiovascular applications [10].

The adoption of polymeric biomaterials in cardiovascular applications has translated into various treatment options that can potentially improve patient outcomes [11]. The usage of polymeric biomaterials ranges from heart valve prostheses, stents, vascular grafts, cardiac patches and pacemakers. Polymeric biomaterials used in biomedical applications have to fulfil certain specifications such as being non-toxic, having the appropriate biomechanical properties, suitable biocompatibility and hemocompatibility. In the case of biodegradable polymeric biomaterials, additional considerations would have to be in place, such as the relation of mechanical strength to the degradation rate, and also the toxicity and clearance pathway of by-products produced after degradation in the body [12,13]. Given the detrimental implications that may arise due to poor polymeric choice, several important parameters need to be considered in order to determine the most suitable polymer type for use. In the next three subsections, the background, material considerations for choice of polymer and a brief overview on the progress will be discussed for bioresorbable coronary stents, vascular grafts and cardiac patches.

## 2. Bioresorbable Stents

### 2.1. Development of Coronary Stents

Balloon angioplasty, commonly referred to as plain old balloon angioplasty (POBA), transformed the treatment of coronary artery disease by allowing cardiologists to modify obstructing coronary plaque, restoring blood flow to the heart without invasive surgical procedures [14,15]. However, the efficacy of POBA was diminished by the occurrence of acute vessel recoil or vessel dissection and late onset complications like late vascular remodelling caused by neointimal hyperplasia [16]. Coronary stents were first developed in the mid-1980s, with the intention of providing lasting vessel patency after balloon angioplasty treatment [16]. The Palmaz-Schatz^®^ (Johnson & Johnson) bare metal stent (BMS) was the first FDA-approved balloon-expandable stent developed by Schatz and co-workers [17]. The Belgium Netherlands Stent Arterial Revascularisation Therapies Study (BENESTENT) and the North American Stent Restenosis Study (STRESS) were two pivotal clinical trials in influencing the decision to adopt BMS as a standard care practice, demonstrating clear superiority of BMS over POBA [18,19]. However, the long term follow up of BMS treatment highlighted in-stent restenosis (ISR) rates as high as 20–30%, which contributed to significant morbidity or mortality [20].

Drug eluting stents (DES) were subsequently developed to combat the high levels of ISR experienced by BMS implantation [21]. The first generation of DES was the combination of the metallic stent platform with a durable polymer coating together with an antiproliferative agent [21]. These DES were found to increase the risk of late stent thrombosis (ST) due to delayed healing of the endothelial layer of the vessel and sensitivity to the polymer coating on the DES [21,22]. In the second generation of DES, the choice of chromium-cobalt alloy allowed stent strut thickness to be thinner (80–90 μm), compared to 316 L stainless steel used in first generation DES (130–140 μm) [21]. The thinner struts allowed for more rapid re-endothelialisation of the stent [23], reducing the risk of ST. The second generation of DES also explored the use of other -limus-based drugs such as everolimus and zotarolimus in order to reduce the rate of ST and encourage a more rapid re-endothelialisation [21]. Through drug-polymer matrix modifications and thinner strut designs, the second-generation DES has led to significant improvements in efficacy and safety in PCI treatment.

While the second-generation DES has been largely successful in producing favourable treatment outcomes, certain complications still persist, such as very late stent thrombosis, loss of vasomotion and delayed vessel healing [24]. This led to the development of bioresorbable scaffolds (BRS) that could circumvent such complications. In theory, the complete absorption of the BRS would mean that repeated revascularisation to the same site can be possible with restored vasomotion [16] while leaving nothing behind. This can potentially promote positive remodelling of the artery and eliminate the issue of permanent caging of the vessel [25].

To design a BRS, there are stringent criteria to be met. Ideally, the period for BRS to provide support in a vessel is postulated to be around six to nine months [26,27]. As the BRS degrades, the degradation by-products should not illicit any local or systemic toxicity responses [28]. It should be hemocompatible to reduce the occurrence of thrombus formation and have good mechanical properties to prevent elastic recoil.

Various R&D efforts have explored polymers such as poly (L-lactic acid) (PLLA), polyglycolic acid (PGA), poly [(d,l-lactide)-*co*-(glycolic acid)] copolymer (PDLGA) and polycaprolactone (PCL) [29]. These polymeric BRS face the challenge of having inferior mechanical properties, which necessitated thicker stent struts that eventually led to unfavourable flow disturbances and other deployment issues [30].

### 2.2. Polymers in BRS Application

It is well understood that BRS made from polymers have lower mechanical strength compared to their metallic counterparts [25]. The radial strength of a stent is directly proportional to its tensile modulus, and BRS made of PLLA are about 100-fold weaker than stents made from (biostable) stainless steel and cobalt chromium. To compensate for this intrinsic material shortcoming, strut thickness of these BRS have to increase by 240% [27]. Table 1 shows the physical and mechanical properties of permanent metals and polyester-based materials used in stent and BRS fabrication.

Polyester-based polymers such as polylactic acid (PLA) have a side methyl group chemically linked to the main chain. Though they have the same chemical composition, PDLLA is amorphous while PLLA is considered semi-crystalline (up to ~70%). This variance in crystallinity contributes to a difference in their mechanical properties. Polyglycolic acid (PGA), on the other hand, lacks the additional methyl group and therefore is more susceptible to hydrolysis, due to lower steric hindrance. On the other hand, the lack of a methyl side group allows PGA polymer chains to pack better together and this leads to higher crystallinity, melting temperature and mechanical properties. Polycaprolactone (PCL) is another linear thermoplastic polymer which has a longer repeat unit, and therefore greater chain flexibility. This polymer has a low T_g_ and T_m_, and exhibits high elongation at break and relatively lower tensile strength compared to PLA and PGA. REVA Medical’s BRS is fabricated from tyrosine derived polycarbonate (PC), which has a different chain length of alkyl ester pendent chains that forms the carbonate copolymer, with the trade name of Tyrocore. It is made up of analogues of amino acid tyrosine (iodinated diphenol) and hydroxy esters (low molecular weight polymer lactic acid). The mechanical properties (strength and ductility) of Tyrocore is contributed by the bulky pendant group within the iodinated diphenol while radiopacity of the polymer is attributed to the iodine, which is chemical bonded to the tyrosine analogue [31].

### 2.3. Degradation Profile of BRS

**Figure 1:**
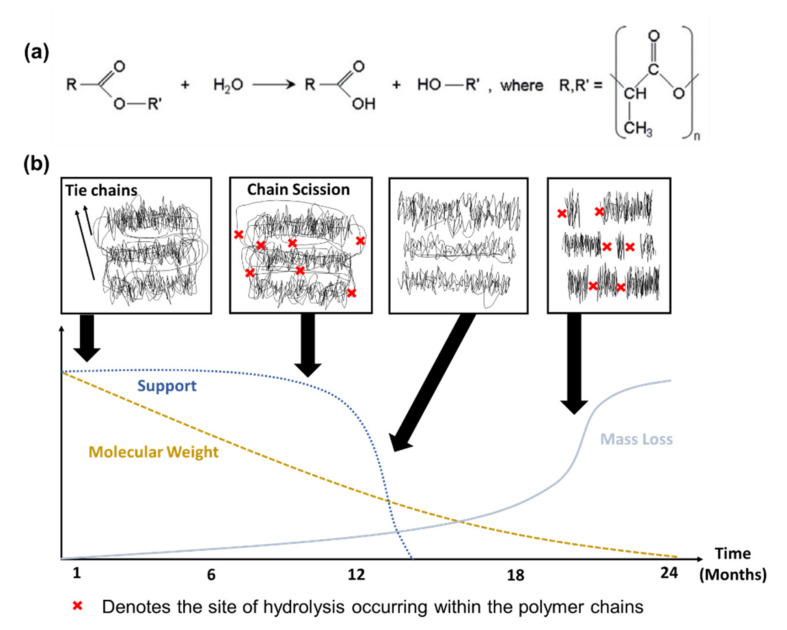
Degradation of aliphatic polylactic acid (PLA). (**a**) Hydrolysis of ester bond. (**b**) Illustration of the loss of device’s radial strength (starts at 6 months), loss of mass (12 months) and molecular weight with time. The complete degradation of BRS is expected to be at 24 months mark [35]. Image adapted from [35].

As most polymeric BRS include aliphatic polyesters, polycarbonates and polyanhydrides, the general degradation mechanisms of these materials are similar—through the hydrolysis of the polymeric chains (Figure 1a) [35]. A schematic representation of the polymer chains during degradation and the corresponding device performance is shown in Figure 1b. PLLA, being a semi-crystalline polymer, has both crystalline and amorphous segments (tie chains). The former has crystals in regular arrangement while the latter has molecules randomly organised. The amorphous region is more accessible to water uptake, and therefore more susceptible to hydrolysis. Thus, in a semi-crystalline polymer, the initial decrease in molar mass occurs only in the amorphous region and thus the loss in mechanical properties is not significant. The second stage of degradation is characterised by the reduction of radial support due to scission of the amorphous tie chains at the crystal-amorphous interface; some degree of crystalline disorder is also evident at this stage. Structural discontinuities and cracks are present owing to fragmentation of polymeric chains into oligomers [36]. Shorter polymer chains (oligomers) are more prominent in the final stage of degradation, with these oligomers starting to escape the bulk polymer as water-soluble entities. Eventually, the L-lactate monomer converts to pyruvate and is broken down to carbon dioxide and water end-products in the Krebs cycle. It is expected that the localised pH level is lower (acidic) due to acidic by-products from PLLA [37]. Completion of the bioresorption of the polymers is effected with the excretion of the end-products through the lungs or kidneys [25,36].

### 2.4. Processing Methods

**Figure 2:**
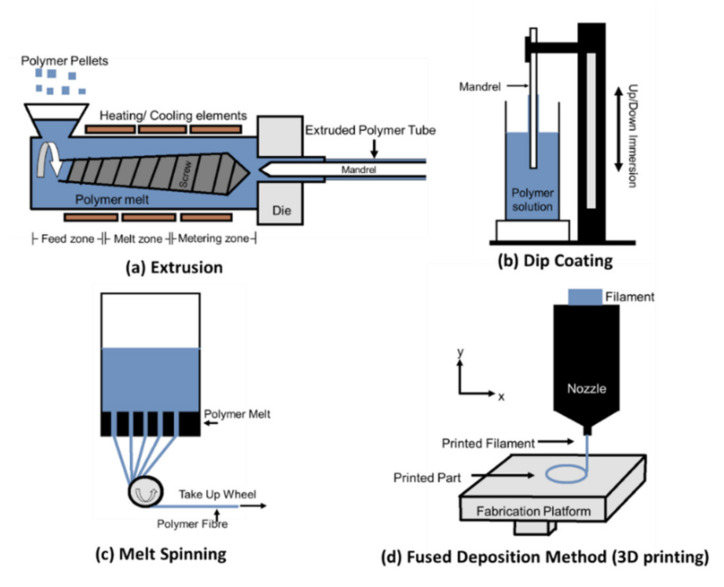
BRS fabrication techniques by (**a**) extrusion and passing the melted polymer through a solid mandrel; (**b**) dip coating of the mandrel in a polymer solution, (**c**) melt spinning and drawing of fibre in aligned orientation and (**d**) fused deposition method (FDM) to deposit material according to the CAD input. Images adapted from [39,40,41].

In the fabrication of polymeric BRS, the solid polymer pellets are first converted into a molten dissolved state before being extruded into a tubular structure. Post processing steps may be performed in order to improve the device’s mechanical properties prior to conventional laser cutting to the desired final scaffold design [38]. The processing method plays a crucial role in influencing the device’s material properties and subsequent mechanical performance. Several methods such as extrusion, dip coating, melt spinning, and fused deposition methods have been employed in the production and post processing of polymeric BRS (Figure 2).

#### 2.4.1. Extrusion

Extrusion of a polymer tube involves melting of polymer pellets in an extruder in the presence of a mandrel. Polymer pellets are first introduced into the hopper of the extruder where it will exit as solid pellets in the feed zone. The polymer is directed toward the melting zone by the extruder’s screw and subsequently to the metering zone where the mandrel is attached to the die. The polymer tube exits the die and is then cooled by either air or water (Figure 2a). The process of extrusion is a continuous whereby polymer is drawn through the die, producing aligned polymeric chain and resulting in anisotropy. This helps in improving the tensile modulus and strength of the tube as the crystallinity increased when the polymeric chains are orientated in an ordered manner [39]. With higher crystallinity, the radial strength of the BRS is improved. Higher crystallinity means a greater resistance to penetration of bodily fluid and hence delaying the degradation process of the device. For example, Abbott’s BVS is first fabricated by extrusion of PLLA resins to form tubes, followed by laser cutting to its desired design. The high crystallinity resulted from extrusion and the thick stent strut (150 μm) contributed to the long degradation of 24 months [30,39].

#### 2.4.2. Dip Coating

This process, as seen in Figure 2b, involves a controlled, repeated immersion and removal of the mandrel into the polymer solution, followed by rotation of the mandrel with the radial direction to eliminate the thickness gradient. The concentration of the polymer solution determines the viscosity, which directly affects the thickness of each layer. Different layers can be formed with different material and orientation to obtain a different set of properties. The final tube properties depend on the amount of frequency the mandrel is coated and the time interval between each immersion. Subsequent post-processing, such as annealing of the product can improve its strength through increasing crystallinity and removal of any residual strains or stresses within the polymer [39,42]. For the formation of the tubular structure through dip coating, the ratio of crystalline to amorphous phases can be tailored in order to enhance radial strength and dimensional stability. This directly correlates to a controllable degradation time and mechanical properties with the manipulation of crystalline phases. Amaranth Medical employed dip coating of high molecular weight PLLA to fabricate their BRS. The APTITUDE BRS, despite a strut thickness of 115 μm, has a resorption time of 3 to 6 months [43]. The MAGNITUDE BRS fabricated by this means is able to achieve a sub 100 μm strut diameter while having comparable radial properties to permanent metallic stents, and the capability of having an overexpansion limit up to 2.5 mm [44,45].

#### 2.4.3. Spinning and Braiding

Wet spinning, dry spinning and melt spinning are similar processes of spinning where the polymers in a melt or solution form, passes through an orifice in a spinneret and is subjected to drawing to align the chain to promote anisotropy (Figure 2c). Fibres spun by wet spinning and dry spinning are spun with non-volatile and volatile solvent while fibres spun by melt spinning are first melted prior to extrusion [46]. Similarly, electrospinning can produce nanofibers through an electrostatic-driven jet where the charged jet of polymer is collected onto a grounded substrate or a rotating drum. Fibres collected in the latter are in an uniaxial direction when subjected to a rotating drum [39]. These fibres can be subsequently woven, knitted or braided into stent-like structure [47]. PLLA filaments prepared by melt spinning can exhibit excellent tensile modulus and strength, flexibility and ductility with fibre drawing as post-processing. The MIRAGE BRS (Manli Cardiology) is fabricated by braiding microfibers to form a helix coil structure that exhibit flexibility and high strength. The braided structure has a low crossing profile (0.044–0.058 in) which aided in delivery [48]. Additionally, melt spun fibres with higher draw ratio (8:1) are reported to have a higher crystallinity than fibres drawn at a lower draw ratio (3:1) which relates to higher tensile modulus and strength. Expandable stents are constructed by looping the fibres in a four-loop configuration, and they have demonstrated good initial radial compression strength up to 8 weeks [49].

#### 2.4.4. 3D Printing

3D printing has been explored as an alternative method in the fabrication of polymeric BRS. The architecture of a specific vessel can be determined by computed tomography (CT) imaging and followed with a customised computer-aided design (CAD) model of a stent for a specific patient. Based on the digital sketch, a stent is printed using fusion filament fabrication (FFF) or fuse deposition modelling (FDM). The prototype is built as a nozzle which extrudes the stent layer-by-layer (Figure 2d). Various studies were conducted by 3D printing their BRS [50,51,52]. Cabrera et al. fabricated their BRS via FDM technology using thermoplastic co-polyester. The 3D-printed BRS has a comparable radial force and deformation to nitinol stents, demonstrating the use of 3D printing to fabricate BRS might potentially be feasible. Similarly, Guerra et al. [52] 3D-printed their composite BRS with PLA and PCL with approximately 85% to 95% accuracy and under five minutes, and the subsequent BRS was able to exhibit good radial properties.

### 2.5. Current BRS

**Figure 3:**
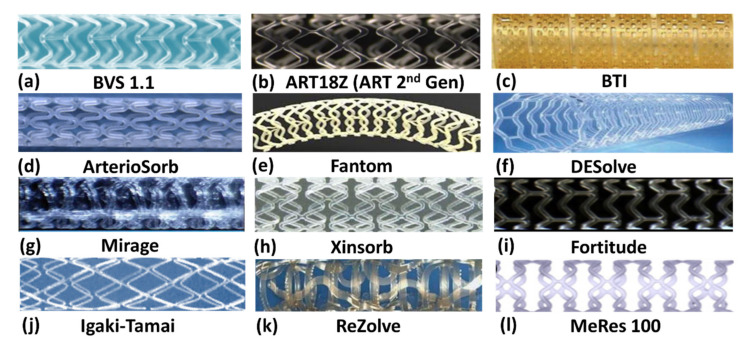
BRS in development, the preclinical or clinical phase. (**a**) Abbott Vascular’s ABSORB BVS 1.1, (**b**) Arterial Remodelling Technologies’ ART18Z (2nd generation), (**c**) Bioabsorbable Therapeutics Inc’s (BTI) stent, (**d**) Arterius’ ArterioSorb, (**e**) REVA Medical’s Fantom, (**f**) Elixir Medical’s DESolve, (**g**) Manli Cardiology’s MIRAGE, (**h**) HuaAn Biotech’s Xinsorb, (**i**) Amaranth’s Fortitude, (**j**) Igaki-Tamai stent, (**k**) REVA Medical ReZolve and (**l**) Meril Life Sciences’ MeRes100 [32,44,53,54].

Most polymeric BRS are made from poly L-lactic acid (PLLA) with the exception of FANTOM BRS (Reva Medical, San Diego, CA, USA), which uses tyrosine polycarbonate (PC). PLLA is a thermoplastic polymer that is widely adopted in biomedical engineering. BRS fabricated from PLLA includes ABSORB BVS (Abbott Vascular, Santa Clara, CA, USA) (discontinued), APTITUDE/MAGNITUDE (Amaranth Medical, Mountain View, CA, USA), DESolve (Elixir Medical, Sunnyvale, CA, USA) (CE marked), and MeRes100 (Meril Life Science, Vapi, India) (CE marked). The Bioabsorbable Therapeutics Inc’s (BTI) stent is made of poly anhydride ester with salicylic acid. REVA Medical developed a proprietary tyrosine derived PC with iodine chemically grafted onto the main chain which provided radiopacity to the device, FANTOM (CE marked). Besides FANTOM, the rest of the BRS are typically tagged with a radiopaque marker to render visual aid during implantation which is important during tracking and deployment. However, the complete resorption timeframe of these polymers deviates from the ideal situation, partly due to the thick struts in comparison to the metallic stents, which are less than 100 μm. Figure 3 shows some of the coronary BRS design that have been developed and reported over the years.

#### 2.5.1. BRS Clinical Experience

Abbott Vascular’s BVS was the first BRS to be approved by US Food and Drug Administration (FDA) but was shortly withdrawn from the market after the ABSORB III data [55]. The ABSORB III clinical trial (prospective, randomised controlled trial, 2008 patients) demonstrated that target lesion failure (TLF) rate between Absorb BVS and cobalt chromium everolimus-eluting stents (Xience-EES) was higher in the BVS-arm (13.4% + SD vs. 10.4% + SD, *p* = 0.06) at 3-year follow-up. Patients implanted with BVS in their vessels with vessel size < 2.25 mm had a more prevalent scaffold thrombosis (ScT) rate (2.3%) due to larger device footprint caused by the thick struts of the BVS [55].

Learning from the BVS, other companies started to work towards achieving thinner struts for their BRS in order to improve device safety [56]. The MeRes-1 trial (prospective, single-armed, multicentre, 108 patients with de novo coronary artery lesions) was conducted using the MeRes100 BRS (strut thickness = 100 μm) showed promising results with low rate of adverse events (1.87%) and no scaffold thrombosis. The follow-up at two years showed no significant difference in scaffold late lumen loss (LLL) (0.13 ± 0.22 mm vs. 0.24 ± 0.34 mm, *p* = 0.10), reduction in mean lumen area (6.17 ± 1.28 mm^2^ versus 5.47 ± 1.50 mm^2^, *p* = 0.21) between six months and two years. In 2019, it was concluded that the MeRes100 BRS have demonstrated safety and efficacy to maintain lumen patency up to two years. There was also no reported late scaffold thrombosis up to three years follow up and the thinner strut of the device might have led to a quicker endothelialisation and resorption rate [57].

APTITUDE and FORTITUDE BRS (Amaranth Medical, Mountain View, CA, USA) are made of PLLA with strut thickness of 115 μm and 150 μm respectively. The MEND I, MEND II and RENASCENT I trials investigated the performance of the FORTITUDE BRS in moderate to high risk lesions with pre-dilatation prior to stent implantation. A combined angiographic analysis of the three trials reported a LLL of 0.29 ± 0.43 mm and 0.27 ± 0.37 mm as well as diameter stenosis of 14.3 ± 12.0% and 17.5 ± 12.8% for nine months and two years follow up respectively [58]. The RENASCENT II trial (prospective, non-randomised, multicentre, non-inferiority) evaluated patients with single de novo coronary lesions undergoing percutaneous coronary intervention with APTITUDE BRS [59]. The study reported an LLL of 0.35 ± 0.33 mm and 0.37 ± 0.44 mm at 9 and 24 months, respectively, which was comparable to the data presented by ABSORB BVS and FORTITUTE BRS. Struts of APTITUDE BRS at nine months were found to be well apposed (96.5 ± 5.02%) and covered with neointimal tissue (97%) despite the continual resorption process. Overall, the RENASCENT II study shown positive data and procedural success rates with no major adverse cardiac events. Shortly after, Amaranth introduced DEFIANCE, with the thinnest ever BRS strut thickness of 85 μm. The company commenced the RENASCENT III trial in 2019 and is hopeful that it can improve clinical outcome to the first generation of BRS [58]. The RENASCENT III trial was designed to evaluate the performance and safety of the MAGNITUDE BRS (Amaranth Medical Mountain View, CA, USA) (98 µm) in moderate to high risk lesions (excluding severely calcified lesions). Nine month angiographic analysis of the trial showed a late lumen loss of 0.28 ± 0.36 mm and diameter stenosis of 13.5 ± 12.5% [60].

The NeoVas BRS (Lepu Medical Technology, Beijing, China) is made from PLLA with a strut thickness of 160μm, has been approved by CFDA. The NeoVas BRS was evaluated for its safety and efficacy in comparison to a metallic DES (CoCr-EES) in the NeoVas Bioresorbable Coronary Scaffold Randomised Controlled Trial (prospective, single-blind, multicentre, randomised controlled trial). 12 months follow-up showed that in-segment lumen loss (LL) and the recurrent rate of angina over one year between NeoVas and CoCr-EES were 0.14 ± 0.36 mm versus 0.11 ± 0.34 mm and 27.9% versus. 32.1% (*p* = 0.26) respectively. The clinical data suggested the clinical performance of the NeoVas BRS were comparable to the DES at 12 months [61].

#### 2.5.2. Looking Forward: Polymeric BRS

While the concept of a transient BRS is attractive, current BRS faces critical issues of large strut thickness and insufficient radial strength, which leads to safety and procedural difficulties. Since the withdrawal of ABSORB BVS, the BRS market has taken a hit, with physicians demanding more long-term safety and efficacy data to justify the cost and risk associated with using BRS over the gold-standard DES. While it is not expected that a polymeric BRS will be able to match the mechanical performance of a metallic stent, improving the material properties of the polymer may potentially address some of the limitations. Novel polymeric composites, processing techniques and designs may help to improve the properties of BRS. Ang et al. [61] reported a novel polymeric nanocomposite platform with the incorporation of functionalised barium sulphate (BaSO_4_) nanofiller to improve PLLA’s mechanical properties. Modelling using finite element analysis showed that the formulation with improved mechanical properties allowed for thinner struts (~100 μm) with better expansion and fracture properties. The novel material was also radiopaque due to the addition of nanofillers.

On top of improving the choice of polymer, it is pertinent to understand the degradation behaviour of polymeric BRS under physiological conditions. It has been reported that when non-uniform degradation takes place, it can affect the integrity of the polymeric structure during the breakdown. Some regions will tend to degrade quicker and cause large deformation which result in flow disturbance and lead to scaffold thrombosis [62]. The penetration of the discontinued struts into the lumen can cause malapposition and activate the coagulation process of thrombus [63]. In addition, the degradation of the polymeric BRS might decrease the localised pH levels of the artery, leading to the recruitment of inflammatory cells [64].

Recently, Biotronik AG’s magnesium-based BRS (Magmaris, 1st generation strut thickness = 150 μm) has demonstrated the potential of magnesium-based BRS. Magmaris demonstrated 86% less platelet coverage compared to contemporary polymeric BRSs and a shorter complete resorption rate at 12 months [65]. The data suggested that the use of a resorbable metallic-BRS might result in a more acceptable thrombogenic and inflammatory response after implantation, thereby improving the safety profile of the BRS.

## 3. Vascular Grafts

Vascular grafting, also known as coronary artery bypass grafting (CABG), involves the use of a conduit to bypass an occluded or diseased vessel in order to restore blood flow to the ischemic region [66]. Although PCI has become the gold standard in the treatment of diseased vessels, CABG have reported superior long term vessel patency [67,68,69]. Preferred conduits for vascular grafting are usually from an autologous source, such as the internal thoracic artery, radial artery or saphenous vein [70]. The saphenous vein remains the most popular choice as a bypass graft due to reduced complications associated with its removal and the ability to remain patent for at least 10 years [70]. In addition to autologous grafts, synthetic grafts made from polytetrafluoroethylene (PTFE) are also an acceptable alternative to autologous grafts [71].

The use of synthetic grafts in large-diameter vessels (>8 mm) such as the iliac arteries has a long-term patency rate of around 90% [72] and has demonstrated favourable patency levels in medium diameter vessels (6–8 mm). However, the use of synthetic grafts in small vessels (<6 mm) has resulted in poorer patency levels compared to the use of autologous grafts. PTFE grafts used in CABG resulted in 1-year patency of around 60% compared to over 95% for the saphenous vein graft [73,74] and for the 2-year patency levels, PTFE grafts declined to 32%, while the saphenous vein graft remained at around 90% [73,74]. Other issues such as patient suitability, quality of vessel or complications during vessel removal remains an impediment to the adoption of CABG techniques for CAD treatment. Moreover, graft failure is not uncommon, especially with the use of synthetic grafts [75,76]. Common modes of graft failure include thrombosis, atherosclerosis, infections or neointimal hyperplasia [76].

There have been efforts to improve the patency of synthetic grafts through seeding endothelial cells on the luminal surface of the graft [77]. Although the improvements made were insufficient compared to natural grafts, this approach highlighted the potential of a tissue engineered vascular graft for future clinical adoption [66,78]. The concept of producing a tissue engineered vessel capable of functioning like a natural graft remains a highly attractive solution to the current limitations faced by finding a suitable graft for patients where autologous sources are not available.

### 3.1. Polymers for Bioresorbable Vascular Graft Scaffold Application

Similar to BRS fabrication, scaffolds for coronary grafts can be fabricated using semi-crystalline synthetic polymers such as PLLA, PCL, PGA, thermoplastic polyurethane (TPU), poly (ester urethane) urea (PEUU), poly (glycerol sebacate) (PGS), poly (*p*-dioxanone) (PDO) or natural polymers like polyhydroxyalkanoates (PHA), naturally derived polymers or decellularised matrix [79]. PHAs are created through the fermentation of food-based carbon sources like sugar and lipids and display tailorable mechanical and degradation properties, depending on their molar mass and synthesis [80,81]. Examples of PHAs includes poly (3-hydroxybutyrate) (PHB), poly (3-hydroxyvalerate) (PHV), poly (3-hydroxyoctanoate) (PHO), poly (3-hydroxynonanoate) (PHN) and their copolymers [82].

Naturally derived polymers consisting of collagen, elastin, fibrin, fibronectin and gelatin form the components of the different arterial layers [83]. These polymers mimic the native ECM which can promote ingrowth of vascular cells [83]. A major ECM component is type I collagen, where the collagen fibres serve to limit large amount of strains, providing stiffness and support required for the integrity of the blood vessel. Collagen was reported to have low antigenic and inflammatory responses and favourable cell attachment during fibrillogenesis [84,85]. Similar to collagen, elastin is another major constituent of the blood vessel ECM and it accounts for the structure’s elasticity. Crosslinked elastin fibres within the vessel prevents recoil of the vessel and any permanent deformation during pulsatile flow. Additionally, the production of smooth muscle cells (SMC) is regulated to prevent neointimal proliferation [86]. Other naturally derived materials to produce bioresorbable vascular graft scaffold include chitosan and silk-derived fibroin. Chitosan is a polysaccharide obtained from chitin, with crustaceans being the main source. Being biocompatible and biodegradable, it is widely used in medical applications such as for antimicrobial purposes and wound healing [87]. Silk, naturally obtained from silkworms, is a biomaterial that is versatile in terms of their physical properties, biodegradability and biocompatibility [88]. Synthetic and natural materials have their respective benefits and shortcomings, a combination of both to optimise the overall properties of a hybrid material can result in a suitable vascular graft.

### 3.2. Strategies and Approaches for a Bioresorbable Vascular Graft Scaffold

**Figure 4:**
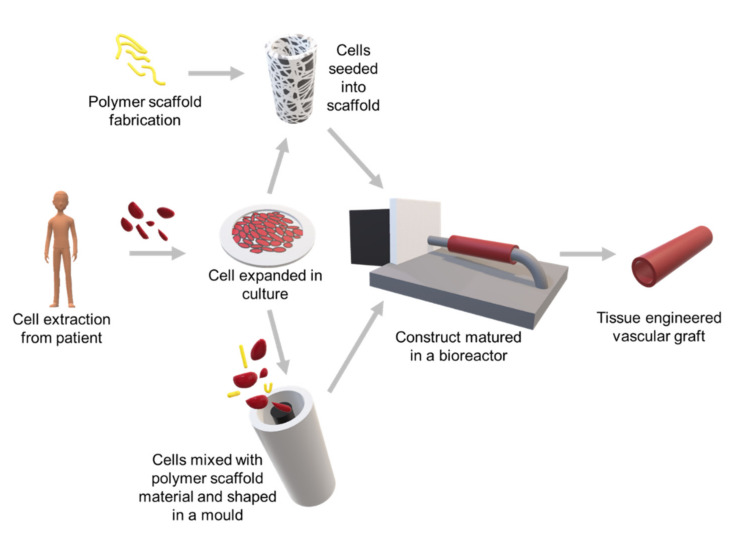
The process of producing a tissue engineered vascular graft through scaffold-based method. The patient’s cells are harvested, then isolated and cultured in vitro before seeded into a scaffold or mixed together with a polymer scaffold material in a tubular mould. Additional conditioning and culturing in a bioreactor is required. Image adapted from [66].

There are various approaches to creating a tissue engineered vessel graft. One conventional way is through a scaffold-based method (Figure 4), with the scaffold being fabricated using either synthetic, natural or hybrid materials. Cells are first extracted and isolated from the patient and subsequently proliferated in culture. The cells can either be seeded directly onto the scaffold or mixed directly with the scaffold material before being assembled into the shape of an artery. The construct will then be transferred to the bioreactor for further stimulation and culture in a 3D fashion. Upon mauration, this cell-seeded 3D construct can ideally be used as a tissue engineered vascular graft [66].

Other methods include the use of decellularised natural matrices and self-assembly techniques to build the tissue engineered vascular graft. The use of decellularised natural matrices derived from animals leverages on the structure and mechanical properties of natural ECM. Decellularisation is a process of removing cellular material through physical means such as abrasion, pressure and agitation or chemical means using acid/bases, solvents and other biological agents [89]. It is important to ensure that these harsh treatments do not destroy the architecture of the ECM.

The self-assembly method can be achieved with various techniques such as 2D sheet-based tissue engineered constructs, cell bioprinting and microtissue aggregation [90]. In the 2D sheet-based method, culturing of cells into sheets is performed prior to stacking them together to form layers. This is followed by shaping these layers around a mandrel, rolling them into a tubular structure before growing the construct into a vascular graft. During the process, large amounts of cell culture media are necessary to perfuse the ECM in order to produce strong and healthy sheets for fabrication. Self-assembly by microtissue aggregation involves depositing droplets of aggregated vascular cells bounded by secreted ECM into a tubular mould, before maturing into a functional structure. Similarly, the cells can be 3D printed precisely and further cultured in a bioreactor. Vessels that are 3D printed has a potential to be produced into complex structures like bifurcations. In this paper, we will focus more on the scaffold-based approach and manufacturing of the polymeric scaffolds.

### 3.3. Fabrication of Bioresorbable Vascular Graft Scaffolds

**Figure 5:**
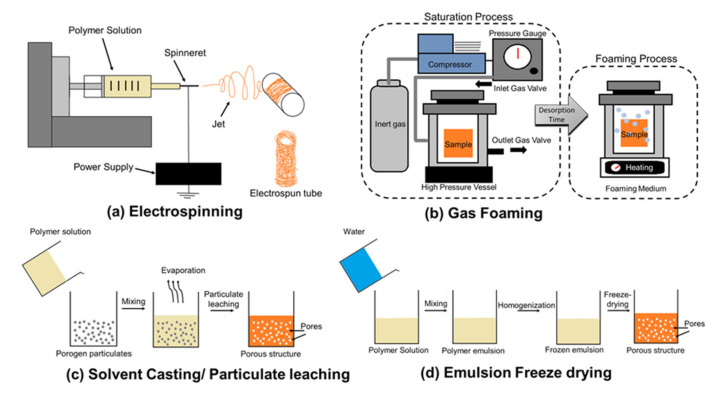
Schematic illustrating methods for scaffold fabrication. (**a**) Electrospinning of mesh onto rotating mandrel, (**b**) Introducing blowing agent with temperature in the process of gas foaming, (**c**) solvent casting followed by particulate leaching and (**d**) emulsion freeze drying to form porous scaffolds. Image adapted from [97].

The popular approach of obtaining a tissue engineered graft is through the scaffold-guided method; allowing the cells to be seeded and moulded into the shape of the vessel [91]. The first step begins with manufacturing of the bioresorbable scaffold with the chosen materials and methodologies to produce scaffold with optimised physical (mechanical properties, pores, degradation kinetics) and biological properties (cell-material interaction). These manufacturing techniques includes electrospinning, solvent casting/particulate leaching, gas foaming, emulsion freeze drying, and thermal-induced phase separation [66] as seen in Figure 5.

#### 3.3.1. Electrospinning

Electrospinning occurs through an electrostatic-driven jet which stretches the viscoelastic/polymer solution into nano/micro fibres and deposit them along the rotating mandrel. The physical characteristic of the scaffold such as dimension, composition, orientation of fibres, pore size distribution and architecture can be well-controlled by this technique. The diameter of the nano/micro fibres, as well as the direction in which it is collected, will influence the mechanical properties and the cell-material interaction. The anisotropic orientation of cells within the blood vessel can be recreated through seeding of cells onto electrospun scaffolds. Zhu et al. [92] fabricated a fibrin-coated PCL scaffold, showing improved cellular attachment of SMCs along the direction of the fibres. In another study by Zhu et al. [93], it was observed that the proliferation and growth of endothelial cells (ECs) were influenced by the modification of collagen on the surface of PLLA-co-PCL mesh to an aligned direction. A co-electrospun bilayer scaffold can be fabricated with the outer and inner layers containing larger and small pores respectively. The larger pores facilitated SMCs infiltration while the small pores recruited the attachment of ECs [94]. Fiqrianti et al. [95] produced a vascular conduit using electrospun PLLA/chitosan/collagen fibers and reported a burst pressure of 2593 mmHg and tensile strength of 2.13 MPa, which are found to be within the range of a native blood vessel. The conduit had a low cytotoxicity (86.2%) and haemolysis percentage (1.04%), suggesting good biocompatibility. Marcolin et al. [96]’s electrospun silk fibroin-gelatin conduit achieved viscoelastic properties close to the native arteries and exhibit cytocompatibility through the spread of fibroblasts. The group reported a burst pressure of (1279 ± 317 mmHg) for their conduit which was comparable to the native saphenous vein’s burst pressure of ~1600 mmHg used in CABG.

#### 3.3.2. Gas Foaming

A 3D porous scaffold is required to support the exchange of nutrients and waste produced locally within the microenvironment as well as to facilitate the growth, proliferation, differentiation and migration of cells. One way to fabricate a 3D porous structure is by gas foaming, whereby polymer foams are produced when the solid phase (polymer) and a gas phase (blowing agent) are mixed together [98]. This technique uses high temperature, organic solvents and relatively inert gas to pressurise the bioresorbable polymer until it is saturated into bubbles to form foams [99]. The challenge of this approach is controlling the sizes of the pores and pore connectivity despite high levels of porosity (>93%) [100,101]. Harris et al. employed a combination of gas foaming and particulate leaching methodology to fabricate a PDLGA matrix with well controlled pore size and connectedness. In vitro data showed that seeded SMCs were well adhered onto the matrix and formed 3D tissues in the scaffold [102]. In another study, a vascular graft made of elastin was fabricated by gas foaming/leaching. It yielded an average pore size of 33 μm that allows the access of endothelial cells to be adhered and proliferate, depositing a confluent layer in vitro [103].

#### 3.3.3. Solvent Casting/Particulate Leaching

Solvent casting/particulate leaching is another conventional method to produce 3D scaffolds. A polymer solution is mixed with well-dispersed particulates of definite diameter, which will lead to the size of the pores form within the scaffold. These particulates (e.g., salt particles), termed porogens, are contained within the structure after the solvent is completely evaporated. The next step will involve the leaching of these particulates/porogens using a suitable solvent in order to form the pores. The benefit of this technique allows pore sizes and porosity to be controlled. Behr et al. [104] fabricated small diameter conduits produced through dip coating of poly (lactide-*co*-caprolactone) (PLCL) (matrix) in the presence of polyvinyl alcohol (PVA) and polyethylene glycol (PEG) (porogen). The structure is subsequently submerged in water to dissolve the PEG, forming pore sizes ranging from 2 to 9 μm. Conduits fabricated through this method are compliant (1.56% ± 0.31 mmHg^−2^) and close to porcine aortic branch arteries (1.56% ± 0.43 mmHg^−2^). In vivo implantation in a rabbit aorta bypass angiogram revealed continuous blood flow, no leakage, absence of aneurysmal dilatation or acute thrombosis for 3 h which suggest potential for clinical applications. Badhe et al. [105] also fabricated a composite chitosan-gelatin microporous hydrogel-based scaffold with bilayer structure to mimic the native artery. The resultant scaffold was highly porous (82% porosity) and had a reported tensile strength of 95.81 kPa with 112.5% ductility. Human dermal fibroblast was seeded in vitro and were able to proliferate over 20 days, suggesting that the scaffold may be a promising candidate in blood vessel tissue engineering.

#### 3.3.4. Emulsion Freeze Drying

In this process, the emulsion mixture is quenched below the freezing point to detain the liquid phase construction, with removal of the solvents by freeze-drying, leaving a solid scaffold with interconnected pores [97]. Through this approach, porosity of up to 90% can be achieved, with pore sizes ranging from 20 to 200 μm [106]. Pores obtained via the emulsion step can be determined by the viscosity, concentration of polymer solution and amount of aqueous phase. A higher polymeric concentration would eventually lead to smaller pore size due to higher shear forces imposed during the aqueous phase. Conversely, a reduction in the amount of aqueous phase will also decrease pore sizes. Removal of the aqueous solvent leads to the formation of macropores during homogenisation as the micropores tend to coalesce.

Other parameters such as the quenching rate prior to freeze drying, can also influence the size of the pores achieved. A slower cooling rate can produce larger pores due to the tendency of coalescence as the interfacial energy decreases [107]. Norouzi et al. [108] combined freeze-drying and electrospinning to produce a bilayer heparinised vascular graft with PCL/gelatin-heparin being the inner layer and gelatin hydrogel forming the outer layer. The structure had an average pore size of more than 200 µm and seeded SMCs were able to attach and proliferate. Yuan et al. [109] developed a gelatin-based scaffold through emulsion freeze drying technique and was able to achieve an interconnected porous structure with pore size of ~300 μm with narrow distribution. The reported pore size of 300 μm allows vascular cells to infiltrate into the scaffold for vascularisation [110]. It has also displayed excellent cytocompatibility and hemocompatibility.

#### 3.3.5. Thermal-Induced Phase Separation (TIPS)

Formation of a porous scaffold by TIPS involves cooling of the solution below the solvent’s freezing point to force phase separate into a polymer-rich phase that solidifies and polymer-poor phase that crystallises. The crystals are removed by sublimation through freeze drying leaving behind a porous structure. The porous structure can be controlled by polymer concentration, quenching rate and temperature. Through increasing the polymer concentration, viscosity increases and with a lower amount of solvent, porosity decreases. The viscosity limits the growth of crystal and will lead to the formation of smaller pores. Another factor which governs the size of the pores is the quenching temperature, as smaller crystals tend to nucleate at lower temperature while growth of the crystals occurs at high temperature. Thus, scaffold fabrication under lower quenching temperature yields smaller pore sizes as the crystals do not have time for crystal growth and vice versa [97].

Yang et al. [111] fabricated a highly porous (>90%) PLGA scaffold through a combination of TIPS and particulate leaching, producing pore sizes ranging from 75 to 400 μm. The mechanical properties of the scaffold fabricated by TIPS is governed by the amount of pores within the scaffold. Porosity can be controlled via the polymer concentration, freezing temperature and the volume of particulates present. Mi et al. [112]. developed a triple-layered vascular scaffold by electrospinning and TIPS, using TPU and poly (propylene carbonate) (PPC). The electrospun TPU and PPC (1st and 3rd layer) provided mechanical support while the TIPS TPU middle layer had good porosity and interconnectivity for ECs attachment and proliferation.

### 3.4. Preclinical Studies of Bioresorbable Vascular Graft

Current patency rates of small diameter (<6 mm) vascular bypass are not considered satisfactory. There is a keen interest in developing alternative grafts through the scaffold-based approach [113]. This method is very much dependent on the scaffold’s ability to allow attachment/proliferation of cells while it gradually degrades, allowing the deposition of ECM and for the new tissue to grow and replace the graft. There have been several preclinical studies investigating the feasibility of cell-seeded vascular grafts and a non-exhaustive list can be found in Table 2.

Sugiura et al. [114] developed a PLCL scaffold by freeze drying and reinforced it with electrospun PLLA nanofibers. The bioresorbable grafts were implanted in mice over a period of 8 weeks and showed infiltration of cells into the scaffold, growth of ECs leading to the deposition of collagen and elastin. The vascular scaffold graft showed neotissue formation when placed in an arterial circulation environment. Centola et al. [115] combined an electrospun PLLA scaffold with an exterior FDMed-PCL armour to create a vascular graft, and the PCL layer was found to improve the mechanical properties significantly. The seeding of human mesenchymal stem cells (hMSCs) on this graft showed favourable cell attachment onto fibrillar structure, resulting in tissue formation and differentiation into vascular cells. Alessandrino et al. [116] fabricated a triple-layered silk fibroin scaffold for vascular graft using electrospinning technique to mimic the properties of the fibrous components in the ECM. In vitro studies verified the scaffold’s biocompatibility, cell adhesion, proliferation and metabolic activities when seeded with vascular cells up to 20 days. In another study, Zhang et al. reported a chitosan/gelatin scaffold fabricated through TIPS and freeze drying to yield a vascular scaffold [117]. In vitro SMCs attachment and proliferation rate was rapid and became confluent after 72 h, suggesting that the scaffold can be further modified to culture both SMCs and ECs. Zhao et al. [118] made a hybrid vascular scaffold by electrospinning RGD-recombinant spider silk protein (pNSR32), PCL and chitosan and implanted in a rat and results showed patency of up to 8 weeks. These preclinical studies demonstrated the feasibility of developing reproducible bioresorbable grafts using synthetic and natural polymers with custom-made properties that are critical for a biocompatible vascular graft.

### 3.5. Challenges Ahead: Bioresorbable Vascular Graft

The production of a bioresorbable vascular graft would require the management of many parameters—material choice, surface properties, porosity, scaffold architecture and cell-material interaction. Synthetic, natural or hybrid scaffolds can have favourable cell–material interactions through means of surface modification to improve surface hydrophilicity to encourage cells’ infiltration, adherence and proliferation. Despite advances in the development of artificial bioresorbable grafts, one of the major reasons behind the low patency rate of these implants is compliance mismatch [6,128,129]. In addition, the mismatch between degradation rate of the scaffold and ECM deposition rate will lead to structural failure of the cell-seeded construct. While the convenience of an off-the-shelf graft is alluring, the availability of such grafts is currently hindered by protracted preclinical and clinical developments. Clinical trials such as those by Shin’oka et al. [130] and Breuer et al. (NCT01034007) have demonstrated promising results in using tissue-engineered vascular grafts (TEVGs) [131]. In both clinical trials, TEVGs were implanted into paediatric patients to investigate the long-term safety and viability of the TEVG conduits. In Shin’oka et al.’s study, 23 patients were implanted with TEVG, which acted as an extracardiac total cavopulmonary connection. Long-term follow-up (~5.8 years) indicated that none of the patients had any complications such as aneurysmal dilatation or graft rupture. In addition, 96% of patients could discontinue anticoagulation therapy, in contrast to PTFE graft patients, who would require long term anticoagulation therapy [130]. The clinical trial conducted by Breuer et al. (NCT01034007) was similar in approach, with differences in the TEVG fabrication. Instead of a manual approach of pipetting cells onto the scaffold, the technique was improved to use an operator-independent vacuum seeding technique, which yields a more homogenous seeding pattern [131]. Additionally, standardisations in the cell culture approach and cell concentrations together with validation studies conducted in animals resulted in FDA’s approval to conduct a clinical trial for TEVG use in paediatric cardiothoracic application.

While the trials demonstrate the progress of TEVG, the method of producing the TEVGs used in these clinical trials required ex vivo cell seeding, which severely diminishes the shelf-life of such conduits [131]. Given the urgency of treatment needed in most CVD complications, the extensive process of harvesting and culturing cells to prepare TEVGs is not desirable and is complicated to scale-up. A recent study by Fioretta et al. demonstrated that tissue-engineered heart valves (TEHVs) made from bis-urea-modified polycarbonate (pre-seeded with bone marrow cells (BMCs)) performed worse than the cell-free alternative in chronic in vivo performance [132]. Similar levels of valve pliability and functionality were observed between the cell-free and BMC-seeded TEHV during the acute phase (~4 h) and up to 4 weeks in vivo [133]. However, transoesophageal echocardiography (TEE) at 18 weeks revealed moderate to severe regurgitation for the BMC-seeded TEHV [132]. The compromise in performance for the pre-seeded TEHV could be attributed to the fusion of the leaflets along the commissures as well as calcification on the valve hinges [132]. In contrast, the cell-free TEHV did not exhibit the development of calcified nodules or leaflet fusions along the commissures [132]. The observed differences were further confirmed via gene expression analysis, where genes responsible for the activation of valve interstitial cells (TGF-b1 and IL-6), calcification (B-GLAP and bone morphogenetic protein-2) and matrix remodelling of regurgitant valves (MMP-3 and MMP-9), were more highly expressed in the pre-seeded TEHVs, compared to the cell-free TEHVs [132]. These findings led to the investigators discouraging the pre-seeding of BMCs for bis-urea-modified polycarbonate TEHVs [132].

In conclusion, the findings from the pre-clinical and clinical studies warrants a need for greater understanding of the biological remodelling processes in TEVGs. Further elucidation of the remodelling process in cell-free TEVGs could make clinical adoption of cell-free and off-the-shelf grafts a possibility in the future [131].

## 4. Cardiac Patches

The concept of engineering a cardiac patch is to produce a highly functioning layer of heart tissue in order to restore cardiomyocytes that are lost after an ischemic event [134]. Fabrication of a cardiac patch is possible through the combination of engineering and biological sciences [135], birthing the classical tissue engineering paradigm [135]. The cells of interest are isolated from the patient, seeded onto a suitable scaffold, which mimics the architecture and mechanical properties of the actual tissue [134,135]. The seeded scaffold would then be cultured in a bioartificial system which mimics the microenvironment of the cell source, such as the biochemical, electrical or mechanical stimuli [134]. By providing the optimal conditions similar to the native tissue, the cultured cells would ideally begin to proliferate and produce their own extracellular matrix, eventually maturing to become a highly functioning tissue, capable of performing tissue specific functions [136]. Hence, there have been several attempts to optimise the tissue engineering approach to cultivate cardiac patches that closely resembles the native myocardium [137]. Factors such as the cell line, culture medium and even scaffold material are known to affect the maturation and differentiation of the seeded cells into functional cardiomyocytes [134,137].

**Figure 6:**
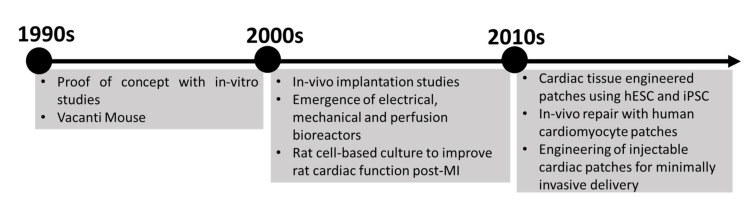
Brief history of TE products leading up to cardiac patches conceptualisation. Adapted from [134].

Figure 6 details the progress made in cardiac patch tissue engineering. In 1997, Eschenhagen et al. [138] demonstrated the feasibility of culturing embryonic chick cardiomyocyte in collagen scaffold to produce a 3D heart tissue with coherent contractions. Following that, in the early 2000s, Zimmermann and colleagues showed that improvements made to such tissue cultures such as the incorporation of mechanical loading during tissue culture and use of co-cultures containing endothelial cells, can vastly improve the morphology and performance of the seeded cardiomyocytes [139,140]. Given the successes of culturing cardiac tissues in vitro, the next obvious step would be to use functional cardiac patches to restore the performance of the heart post-MI. Using fibrin engineered cardiac patches seeded with human induced pluripotent stem cells, Gao et al. was able to improve the left ventricular functions in swine suffering from MI and showed engraftment rates of 10.9 ± 1.8% at 4 weeks [141].

**Figure 7:**
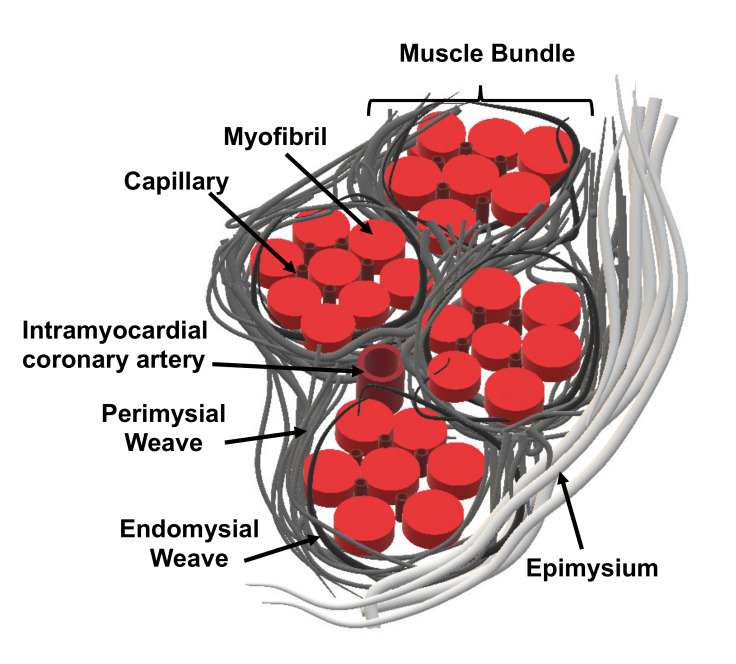
Schematic of cardiac extracellular matrix.

One key aspect of the tissue engineering approach would be the choice of scaffold material, which will directly modulate cell-scaffold interactions. The purpose of the scaffold is to mimic the natural extracellular matrix (ECM) found in the myocardium, and the choice of scaffold material will influence the scaffold’s properties. The natural ECM found in the myocardium comprises mostly fibrillar types I and III collagen, intricately arranged to form the epimysium, perimysium and endomysium [142] as seen in Figure 7. Type I collagen arranges in the form of thick rod like structures and is predominantly found in the epimysium and perimysium, forming a major structural component [143]. Meanwhile, type III collagen is mostly found in the endomysium, as it forms a fine network of collagen fibres that surrounds the myocytes and capillaries [144].

The role of the collagen network is to provide structural support, ventricular geometry and the transmission of force during cardiomyocyte contraction [142]. In addition to the material choice for scaffold fabrication, fabrication technique also plays an important role in replicating the architecture of the native ECM [134]. With careful control of the fabrication technique, anisotropic structures can be created, guiding the orientation of the seeded cardiomyocytes [134]. The suitability of different biomaterial choices for the various fabrication techniques and feasibility in producing a highly functional cardiac tissue patch will be discussed in greater detail in the coming sections.

### 4.1. Commercially Available Cardiac Patches

Conceptually, cardiac patches can be used for the repair of the myocardium; providing structural support and an environment for native myocardial cells to regenerate in. Unfortunately, the post-mitotic status of adult cardiomyocytes would mean that the heart has extremely poor regenerative capability [145]. Hence, the adult heart would be unable to replenish the billions of lost cardiomyocytes after a myocardial infarction (MI) event, even in the presence of a suitable microenvironment [145]. Hence, current commercially available cardiac patches only serve the primary purpose of providing pericardial closure with focus on preventing suture line bleeding [146].

In the list of commercially available cardiac patches presented in Table 3, all of these commercially available cardiac patches are acellular. This could be a reflection on the current limitations faced when attempting to produce a fully functionalised cardiac patch. Moreover, in a follow-up study involving 110 patients implanted with Gore-Tex^®^ surgical membrane, no cellular ingrowth or immunocompetent cellular elements was found on the surgical membrane [147], highlighting the inertness of the chosen surgical membrane. Xenografts based on natural cardiac ECM could become an attractive alternative solution, as it is a better mimic of the host’s cardiac ECM, enabling better cell migration and proliferation [148].

However, naturally derived cardiac ECM are often treated with glutaraldehyde to form crosslinks in the cellular and extra cellular matrix proteins, stabilising the structure and substantially reducing graft immunogenicity [149]. Glutaraldehyde treatment has been found to cause cytotoxicity, early mechanical failure and severe calcification in bovine pericardium [149]. Hence, the shortcomings of existing cardiac patches leaves much to be desired in their capability to provide restorative function in a reproducible manner.

### 4.2. Material Choice for Cardiac Patches

Given the complex architecture of the cardiac ECM, polymeric scaffolds are excellent candidates for the creation of a highly tuneable microenvironment for the proliferation and functionalisation of myocardiocytes [7]. The scaffolds can be manipulated to alter their mechanical strength, degradation profile and even surface chemical composition to better mimic the native tissue [7]. Therefore, the therapeutic combination of a suitable biomaterial and cell source are vital in creating a restorative cardiac patch. The suitability of different biomaterials as well as their associated fabrication techniques and would be further elaborated upon in the coming sections.

#### 4.2.1. Natural Biopolymers

Natural materials are naturally occurring substances that are produced from biological sources, as such they are usually highly compatible with cells and are unlikely to elicit immune responses or cause thrombosis [7]. Especially in the case of decellularised ECM, the microarchitecture is highly preserved, which strongly favours stem cell proliferation and differentiation [7]. Naturally occurring materials that have been used in cardiac patch fabrication include collagen, chitosan, fibrin, alginate, Matrigel, gelatin and decellularised ECM [150]. Benzoni et al. investigated the use of using a chitosan/collagen blend to produce a cardiovascular scaffold [151]. The chitosan/collagen blend followed a 5:1 ratio, and was fabricated via electrophoretic deposition with intersections of cubic and hexagonal rectangular grooves, forming four and six neighbour pores [151]. The choice of incorporating chitosan into the mixture is due to the cationic nature of chitosan which allows it to interact with anionic glycosaminoglycans (GAG) and proteoglycans [152]. While glycosaminoglycan may represent only a small proportion in the non-structural ECM component, their presence in the cardiac ECM is critical in allowing for modification of proteins such as growth factors to occur, altering or adding to their existing functionalities [153]. Furthermore, the fabricated scaffold was designed with 30 µm to 80 µm ridge widths to align the seeded cardiomyocytes, improving their maturation and functionality [154]. To verify the biocompatibility of the chitosan/collagen scaffold, human cardiomyocytes derived from human iPSCs cell lines were seeded onto the scaffold and cultured for 14 days [151]. After 14 days of culture, the cardiomyocytes were found to be exhibiting contraction and expressing functional cardiac genes [151]. Additionally, the effects of the fabricated grooves on cardiomyocyte orientation was investigated by analysing the orientation of Troponin T, a sarcomere protein [151]. It was found that for scaffolds with the patterned grooves, cardiomyocyte proliferation followed the groove direction, in contrast to random cardiomyocyte proliferation in scaffolds without grooves [151]. However, it is worth noting that the production of chitosan requires the stringent processing of chitin (raw material) before it is suitable for use in biomedical applications [155]. Crucial processes includes; demineralisation, deproteinisation, decolourisation and deacetylation [155,156]. Residual proteins, minerals or colour pigments may illicit immune response when implanted in-vivo, especially if the source of chitin was from crustaceans. Hence, fungi may be a better alternative source of chitin as it is less immunogenic, has a narrower molecular mass distribution and can be cultivated to provide a steady source [157].

In another study, by Rashedi et al., the influence of commercially available type-I bovine collagen scaffold on the ability of mesenchymal stromal cells (MSCs) to produce cardiomyocyte markers in a co-culture with rat cardiomyocytes/fibroblasts [158] was investigated. After 5 days of culture, the MSC co-culture in the collagen scaffold began to produce cardiac markers such as MyHC and cTrpT sarcomeric proteins [158]. The level of cardiac markers produced were markedly different compared to a control culture without the collagen scaffold (17.1 ± 1.9% in scaffold vs. 4.8 ± 0.2% in control for MyHC, *p* < 0.01 and 32.9 ± 3.1% in scaffold vs. 5.9 ± 0.7% in control for cTrp-T, *p* < 0.01) [158]. This highlighted the positive impact the microenvironment of an ECM has on stem cell differentiation and viability [7,158]. Hence, with careful consideration for the material processing techniques, the production of a scaffold that closely mimics the native cardiac ECM is possible. This allows for greater success in creating a functional cardiac patch that can restore cardiac functions post-MI.

#### 4.2.2. Synthetic Materials

While the use of natural materials to fabricate cardiac patches is highly attractive due to its chemical composition closely resembling the native ECM, synthetic materials are also a viable alternative material for scaffold fabrication [7]. Synthetic materials are usually readily available with reproducible fabrication processes. Additionally, synthetic materials may offer greater customizability in terms of degradation profile and mechanical strength by controlling the proportions of the individual constituents [7,159]. Synthetic materials such as poly lactic-co-glycolic acid (PLGA), poly ε-caprolactone (PCL) and poly glycerol sebacate (PGS) are commonly used in cardiac tissue engineering [159]. PGS, developed by Robert Langer, is a biodegradable yet elastic polymer produced through polycondensation of glycerol and sebacic acid [160]. The use of PGS in cardiac patch fabrication was investigated by various groups. Tallawi et al. performed a combination of soft lithography and electrospinning techniques to produce a scaffold comprising PGS and PCL polymer in a 2:1 weight ratio [161]. The electrospun PGS/PCL scaffolds are porous in nature, allowing for the exchange of nutrients and waste to occur within the scaffold [161]. In addition, the scaffolds were fabricated with different topographical features such as grooves of 10 µm with 7 µm and 200 µm interspatial distances between the grooves (Different-line), parallel grooves with 7 µm and 10 µm interspatial distance (Parallel-line) and square patterns of size 100 µm with 50 µm between each square pattern (Square) [161]. Using C2C12 cells (immortalised mouse myoblast cell line), cell proliferation and morphology were investigated [161]. It was reported that the seeded cells all exhibited typical myoblast morphology with the appropriate cytoskeleton organisation regardless of the topographical features of their surroundings [161]. However, cell alignment was most prominent in cultures with parallel-line topographical features compared to the multi-directional spreading of cells on a control scaffold made up of random fibres [161]. Additionally, post-natal rat cardiomyocytes were also used observe cardiomyocyte adhesion and alignment [161]. Cell attachments were significantly higher for fibronectin coated scaffolds and glass coverslips compared to gelatin-coated glass coverslips. Cell alignment was most significant (~60%) in scaffolds with parallel grooves and regular interspatial distance [161]. In summary, synthetic materials are readily available with various fabrication techniques that can be applied to achieve the desired scaffold properties. With careful tuning of synthetic material properties, the degradation profile of the scaffold can be made to match the ECM production of the seeded cells, allowing for a complete replacement of the synthetic scaffold over time.

### 4.3. Material Fabrication—3D Bioprinting

The fabrication processes mentioned in Section 3.3 can also be applied similarly to fabricate cardiac patches. With recent advancements in 3D printing, printing of biomaterials such as human cells together with ECM components (bioink) and growth factors to produce a viable cardiac patch is now possible. This method of 3D printing, aptly named bioprinting, allows for a thicker cardiac patch complete with an internal vasculature to be printed, ensuring the cardiac patch’s survivability upon transplantation [162]. The most common problem plaguing most cardiac patches would be the failure to achieve the critical number of cardiomyocytes needed to bring about therapeutic effect upon implantation [7]. The printing process in 3D bioprinting circumvents this issue by layering bioink above one another, creating a 3D structure [7]. This ensures a homogenous distribution of cells throughout the scaffold with a complex porous network that allows for efficient exchange of nutrients and waste, even to cells deep within the scaffold [7].

Nadav et al. reported the successful creation of a 3D printed vascularised cardiac patch using autologous acellular and cellular biomaterials [162]. The cardiac patch was designed based on the CT images of a patient’s coronary network [162]. From the heart CT image, a patch containing the geometry of the major coronary vessel in the region they seek to augment was printed, with microvasculature being added to ensure optimal oxygen distribution to the entire patch [162]. The cell viability of the 3D printed cells matched the viability levels in the cell culture [162], indicating a successful implementation of the 3D bioprinting technique. The group demonstrated that 3D bioprinting is capable of producing a viable cell dense cardiac patch that can potentially be used to replenish the lost cardiomyocytes post-MI.

Bejleri and colleagues reported the bioprinting of cardiac ECM in combination with human cardiac progenitor cells (CPCs) using gelatin methacrylate (GelMA) as the support material [163]. The choice of GelMA as the support material was pivotal to the success in bioprinting natural ECM [163]. The use of support material is essential due to the low viscosity of therapeutic concentrations of natural ECM solutions, which does not allow the printed fibre layers to stack upon each other [164]. While polycaprolactone (PCL) can be employed as a biodegradable support material, the rigid nature of PCL causes a mechanical mismatch with the native ECM making it unsuitable for use as a support material [165]. Hence, the choice of GelMA is able to provide the appropriate mechanical and degradation properties needed for natural ECM bioprinting [163]. The printed bioink (CPCs and ECM) demonstrated high levels of cell viability (~80%) and differentiation (~30 fold increase in gene expression of MYH7) after 6/7 days of culture [163]. The use of bioprinting has shown great promise in producing highly viable and functioning cardiac patches in a reproducible manner.

### 4.4. Future Challenges—The Ideal Cardiac Patch (From a Materials Perspective)

There have been a few cell therapy clinical trials, involving the direct injection of stem cells to the heart to replenish the lost cardiac cells post-MI in an attempt to prevent adverse cardiac remodelling [166]. Despite an improvement in clinical outcomes such as left ventricular ejection fraction (LVEF), cell engraftment rates are low. Therefore, one possible solution is to produce an ECM to provide the cells with a suitable microenvironment for proliferation and differentiation and improve engraftment rates [167]. The cardiac patch is also able to provide physical support to the thinned myocardial wall after an MI event, improving cardiac performance and impeding disease progression of the infarct region [168]. However, the biggest challenge to overcome would be the prevention of arrhythmias when implanting a functional piece of cardiac patch onto the heart due to electrical or mechanical mismatch [169].

In an ideal cardiac patch, there needs to be an incorporation of three distinct cell types—cardiomyocytes, smooth muscle cells and endothelial cells—to provide complementary support to each other. From the material and scaffold perspective, the scaffold compositions have to be able to accommodate and be conducive for the different cell types. If stem cells are used, for example, the scaffold has to provide a microenvironment that closely resembles the native myocardium, allowing for proper differentiation, maturation and alignment of the seeded stem cells. Therefore, the scaffold microenvironment has to contain the necessary physical and biochemical cues for cell adhesion and differentiation as well as a complex porous network to allow for the exchange of nutrients and waste in order to sustain cell viability within the scaffold. Finally, when implanted, the scaffold would have to be able to integrate both mechanically and electrically with the patient’s heart to prevent arrhythmias from occurring [7].

With these criteria met, the cardiac patch would form one of the key solutions necessary in myocardium tissue engineering. The recent conclusion of the ESCORT trial (NCT03676517) in 2018 demonstrated the safety of human ESCs loaded fibrin patch when implanted in patients with severe ischemic left ventricular dysfunction [170]. The ESCORT trial was a phase I clinical trial, which involved the epicardial delivery of a fibrin patch embedded with a median dose of 8.2 million human ESCs in 10 patients during CABG procedure [170]. None of the 10 patients were reported to have developed any complications (e.g., arrhythmias) and had improvements to the left ventricular ejection fraction and wall motion of the patched region [170]. The results of this clinical trial highlighted the progress made towards a successful implementation of cardiac patch, as arrhythmia, a major complication encountered when implementing cardiac patch, has been shown to be resolved to date. The results from anther ongoing trial CARDIOMESH trial (NCT03746938, follow-up due October 2020) would further elucidate the potential of cardiac patches in treating CVDs.

## 5. Conclusions

This review paper has extensively discussed the concepts behind three major applications of bioresorbable materials in the cardiovascular domain, namely BRS, vascular grafts and cardiac patches. The choice of biomaterials used can generally be categorised into natural and synthetic sources, with natural polymers generally being biocompatible for cell support and synthetic polymers possessing greater mechanical strength. Future implementations of biomaterials could involve a hybrid of these two sources in order to engineer specific properties that better suit their intended implantation [11]. Current advances in fabrication, such as 3D bioprinting, have allowed for more complex hybrid structures to be printed, which bodes well for the successful implementation of tissue engineered products [171].

Among the three applications, BRS has made the most, albeit short-lived, progress in terms of clinical adoption. The number of clinical trials conducted by various BRS companies have allowed the medical community to learn thoroughly from their clinical experience. The inherent weaker material properties of using bioresorbable polymers resulted in large profile of the implants, which in turn led to delivery difficulties and unsatisfactory outcomes. There have been no new FDA approvals of any BRS since the withdrawal of the ABSORB BVS in 2017. Future BRS, polymer- or metallic-based, have the monumental task of addressing the long-term safety and efficacy limitations that surfaced during the ABSORB era. For vascular grafts/TEVG and cardiac patches, overcoming challenges related to reproducibility, scaling-up, biocompatibility and storage is crucial to wider clinical adoption. Initial implantations of these TE products in humans have yielded promising outcomes as discussed in this review. More long-term clinical data from well-designed randomised controlled trials will be required to validate their use in CHD treatments.

In conclusion, the concept of using bioresorbable polymeric materials in cardiovascular applications have showcased its versatility and valuable advantages, with several successful examples of in clinical trials (e.g., in TEVG products). The increasing demand for transient biomedical devices and regenerative medicine driven by ageing and affluence will continue to spur the innovation of better bioresorbable polymers as well as better use of these materials. Coupled with the scientific advancements in biomaterials engineering and 3D scaffold fabrication methods, the potential and development in this area is immensely promising.

## Figures and Tables

**Table 1:** Physical and mechanical properties of polymeric BRS materials [32,33,34].

Material	T_g_ (°C)	T_m_ (°C)	Modulus (GPa)	Strength (MPa)	Elongation at Break (%)
SS316L	NA	~1400	193	668	40
Co-Cr	NA	1454	210	235	40
PLA	60	180–190	2–4	65	2–6
PLLA	60–65	175	2–4	60–70	2–6
PDLLA	55	NA	1–3.5	40	1–2
PGA	35–40	225–230	6–7	90–110	1–2
PLGA (82/12)	50	135–145	3.3–3.5	65	2–6
PCL	54	55–60	0.34–0.36	23	700–1000
PC	147	225	2–2.4	55–75	80–150

NA: Not applicable.

**Table 2:** Preclinical Studies Involving Synthetic, Natural and Hybrid Vascular Scaffolds.

Scaffold Materials	Scaffold Type	Technique of Fabrication	In Vitro/In Vivo Findings
PLCL scaffold with reinforced PLA nanofiber [114]	Synthetic	Freeze-drying, electrospinning	In vivo model: rat aortic implantation (8 weeks) Pore sizes: 12.8 ± 1.85 μm, 28.5 ± 5.25 mm Cellular and macrophages infiltration with ECM deposition present
Degradable polar/hydrophobic/ionic (D-PHI) PU scaffold [119]	Synthetic	Particulate leaching	In vitro: Human coronary SMCs (4 weeks) Mechanical properties (Dynamic): 74 ± 9 (Week 0), 128 ± 20 (Week 4) Increase in DNA mass, cell area distribution and coverage and a contractile phenotype
PGS scaffold [120]	Synthetic	Particulate leaching, freeze drying	In vitro: Mouse embryonic fibroblast cells Pore sizes: 5–20 μm, interconnect macropores of 75–150 μm Fibroblast attachment, adhesion and proliferation in 8 days and collagen covering surface
PLLA/PCL scaffold [115]	Synthetic	Electrospinning, fused deposition modelling	In vitro: Human mesenchymal stem cells (hMSCs) Mechanical properties: Ultimate Tensile Strength (UTS): 1.58 ± 0.07 MPa, Strain to failure: 1.78 ± 0.10, Burst pressure: 0.30 ± 0.03 MPa Cell viability of >90%, presence of proliferation and differentiation (positive CD31 expression in cells)
PU/PLLA scaffold [121]	Synthetic	Multistep-dip coating	In vivo model: rat abdominal aorta implantation (12 weeks) Regeneration of SMCs and ECs on the anastomotic side, presence of fibrohistiocytic tissue from the perigraft tissue, formation of neoarteries
PLA/TPU scaffold [122]	Synthetic	TIPS	In vitro: mouse fibroblast cell 10 days) Porosity: 80 to 90% Compressive modulus: 5 MPa, 15% weight loss rate over 4 weeks Fibroblast cells proliferation and migration, favourable biocompatibility
Crosslinked collagen/elastin scaffold [123,124]	Natural	Freeze drying	In vitro: Human SMCs (14 days) Porosity: ~90% Mechanical properties: High (38 ± 2 kPa) and low (8 ± 2 kPa) strain stiffness, yield stress (30 ± 10 kPa) and strain (120 ± 20%) Dynamic condition showed improvement of tissue deposition and more homogenous distribution of SMC, higher collagen mRNA expression levels and therefore proliferation
Silk scaffold [125]	Natural	Gel spinning, freeze drying	In vivo model: rat abdominal aortas (4 weeks) Mechanical properties: Tensile modulus 2.20 ± 0.9 MPa, UTS: 0.273 ± 0.11 MPa Proliferation and migration of SMCs and ECs into the silk graft, confluent endothelium
Fibrin hydrogel microfiber [126]	Natural	Electrospinning	In vitro: Human endothelial colony forming cells (ECFCs), human SMCs, pericytes (11 days) Improved ECM deposition from vascular cells and a perivascular multicellular layer
Chitosan/gelatin scaffold [117]	Natural	TIPS, freeze drying	In vitro: rabbit aorta SMCs (72 h) Porosity: 81.2% Pore size: 50 to 150 μm Mechanical properties: burst strength of 4000 mmHg Proliferation of vascular SMCs, depicting biocompatibility
Elastin scaffold [103]	Natural	Gas foaming, particulate leaching	In vitro: Human SMCs, endothelial progenitor cells (EPCs) (12 days) Average pore size: 33 μm Confluent endothelial layer (4 days), deposition of ECM and cellular infiltration into the vascular graft
PCL/gelatin scaffold [127]	Hybrid	Electrospinning	In vitro: hMSCs (7 days) Contact angle: Hydrophilicity of 26.33 ± 0.45° Favourable cell spreading, proliferation and adhesion
Silk/PCL/Chitosan scaffold [118]	Hybrid	Electrospinning	In vivo: rat abdominal aorta (8 weeks) Seeded of cells onto scaffold: Enhanced attachment proliferation Maintained patency for 8 weeks
TPU/PPC scaffold [112]	Hybrid	Electrospinning, TIPS	In vitro: Human umbilical vein endothelial cells (HUVECs) (2 weeks) Pore size: 20 to 60 μm Interconnectivity of the pores improve coverage of HUVECs, suggested endothelialisation, >85% live cells
PCL/gelatin scaffold [108]	Hybrid	Electrospinning, Freeze-drying	In vitro: HUVECs, rat SMCs (14 days) Mechanical properties: Tensile modulus of 1.55 ± 0.32 MPa, burst pressure: 882 ± 56 mm Hg suture retention: 2.03 ± 0.12 N Endothelial cell attachment with decrease activated platelet adhesion

**Table 3:** List of the brand, material and purpose of commercially available cardiac patches.

Brand	Material	Purpose
CorMatrix Cor™ PATCH	Small Intestinal Submucosa Extra Cellular Matrix (SIS-ECM); Xenograft	Epicardial tissue support and repair
GORE-TEX^®^ Cardiovascular Patch	Expanded Polytetrafluoroethylene (ePTFE)	To cover and support tissue following any injury or degenerative disease
Bard Cardiovascular Patch	Expanded Polytetrafluoroethylene (ePTFE)	Indicated for use in repair and closure of the cardiovascular system.
SteriGraft™—Pericardium	Pericardium Allograft Source	Pericardial defect, dura mater repair, and periodontal reconstruction
CardioCel^®^ cardiovascular bio-scaffold	Acellular collagen sheet prepared from bovine pericardium; Xenograft	Repair of intracardiac defects; septal defects and annular repairs
Cryolife: Cardiac Tissue Matrix/Allograft	Allograft Source	Congenital reconstruction or as buttress material
PB—Bovine Pericardium Patch	Glutaraldehyde Bovine Pericardium; Xenograft	Cardiovascular repair and support

## Abbreviations

BMS	Bare Metal Stent
BRS	Bioresorbable Scaffolds
BTI	Bioabsorbable Therapeutics Inc
CABG	Coronary Artery Bypass Grafting
CAD	Computer-Aided Design
CHD	Coronary Heart Disease
CPCs	Cardiac Progenitor Cells
CT	Computed Tomography
CVD	Cardiovascular Disease
DES	Drug Eluting Stent
ECs	Endothelial Cell
ECFCs	Endothelial Colony Forming Cells
ECM	Extracellular Matrix
FDM	Fuse Deposition Modelling
FFF	Fusion Filament Fabrication
GAG	Glycosaminoglycans
GelMA	Gelatin Methacrylate
hMSCs	Human Mesenchymal Stem Cells
ISR	In-Stent Restenosis
LDLP	Low Density Lipoproteins
LL	Lumen Loss
LLL	Late Lumen Loss
LVEF	Left Ventricular Ejection Fraction
MI	Myocardial Infarction
MSCs	Mesenchymal Stem Cells
PC	Polycarbonate
PCL	Polycaprolactone
PDLGA	Poly [(D, L-lactic-*co*-Glycolic Acid)]
PEG	Polyethylene Glycol
PEUU	Poly(ester Urethane) Urea
PGA	Polyglycolic Acid
PGS	Poly (glycerol sebacate)
PLA	Polylactic Acid
PLLA	Poly L-lactic Acid
POBA	Plain Old Balloon Angioplasty
PPC	Propylene Carbonate
PTFE	Polytetrafluoroethylene
ScT	Scaffold Thrombosis
ST	Stent Thrombosis
TEVG	Tissue Engineered Vascular Grafts
TIPS	Thermal-Induced Phase Separation
TLF	Target Lesion Failure
TPU	Thermoplastic Polyurethane
UTS	Ultimate Tensile Strength
